# Sociocultural factors associated with fish consumption in Lake Albert fishing community: Guidelines for lead and mercury

**DOI:** 10.1080/23311843.2017.1304604

**Published:** 2017-03-21

**Authors:** Andrew Tamale, Francis Ejobi, Charles Muyanja, Irene Naigaga, Jessica Nakavuma, Charles Kato Drago, Deborah Ruth Amulen

**Affiliations:** 1 College of Veterinary Medicine, Animal Resources, and Biosecurity, Makerere University, P.O. Box 7062, Kampala, Uganda; 2 Department of Food Technology and Nutrition, Makerere University, P.O. Box 7062, Kampala, Uganda; 3 Department of Crop Protection, Gent University, Gent, Belgium; 4 Norges teknisk-naturvitenskapelige universitet, Norway

**Keywords:** heavy metal, lead, mercury, fish consumption advisory, vulnerable, community, lifestyle, fish, Lake Albert, developing country

## Abstract

Fish consumption in subsistence fishing community is a life style associated with lead and mercury uptake for humans. Fish consumption is influenced by sociocultural factors, exposure and health risks. Unfortunately, no sociocultural study in the Lake Albert fishing community in light of lead and mercury exists. A cross-sectional sociocultural study was carried out between March and June 2015. A total of 270 household heads in four landing sites in Hoima district completed structured questionnaires and data analyzed using SPSS version 20. The majority of the households (74.8%) had primary education or below, 51.1% drank unboiled water, and 30% perceived lake water safe for drinking. Children under five ate soup (15%) and middle piece of the fish (29%). The Poisson general linear model predicting weekly fish consumption amounts against sociocultural factors showed that household size (*p* = 0.047), male child presence (*p* = 0.007), methods of preparation i.e. salting (*p* < 0.0001), fish parts consumed by adults (*p* < 0.0001), fish preference (*p* < 0.0001), awareness about the beach management unit (*p* < 0.0001), and income from charcoal selling (*p* < 0.0001) were positive predictors. The negative predictors of weekly fish consumption amounts were awareness about fish consumption benefits (*p* < 0.0001), eating young fish (*p* = 0.002), donor agency presence (*p* < 0.0001), and frying as the method of fish preparation (*p* = 0.002). In conclusion, knowledge of the sociocultural factors associated with fish consumption determines the amounts and frequency of the predominant fish eaten. Therefore, to establish and adopt fish consumption guidelines for lead and mercury in the Lake Albert, the sociocultural factors should be integrated in the message disseminated.

## Notes Public Interest Statement

Fish eating is common around fishing communities. However, there is no information about the common fish eaten, the amounts and frequency of consumption. The current study set out to provide this information in the Lake Albert fishing community. The study found out that many homes drank unboiled water and the reason this was done was because it was safe. The factors which increase fish consumption in the Lake Albert are large sized families, methods of storage of the fish and other sources of income. The fish consumption was reduced due to knowledge associated with benefits and risks associated with eating fish, people who eat young fish and some cooking methods i.e. frying the fish. This information is important to fishing communities and government especially when explaining the benefits and risks associated with fish consumption through fish consumption guidelines.

## 1. Introduction

The dilemma involving fish consumption, benefits and risks, and interventions is a whole new area of public health. Therefore, researchers have studied hazards in the fish and its environment, guideline values and health risk for its consumers Taylor and Baumert (2014). The guidelines for fish consumption are called fish consumption advisories (Burger & Gochfeld, 2008). Consumption advisory aim to reduce the risks passed to the vulnerable groups i.e. pregnant women, children less than 17 years, and females of childbearing age through fish consumption (Arakawa, Yoshinaga, Okamura, Nakai, & Satoh, 2006; Chen et al., 2014; Drescher, Dewailly, Sandy, & Forde, 2014; Tuakuila, Kabamba, Mata, & Mata, 2014).

The sociocultural dimensions of a fishing community include: lifestyle and behavior, level of awareness about fish related risks, location of the community, consumption data, activities carried out in the community and individual factors. The lifestyle of fishing communities worldwide exhibits the notion that every activity on the lake is hinged around fishing (FAO/WHO, 2011). This lifestyle is presented and observed in the diet, boat making, livelihood, cultural norms and social networks build around the community. Failure to recognize the food sharing lifestyle of the Tongo community led to failure of adoption of fish advisory guidelines (Bender, 2007). Communities continued to consume the highly contaminated fish in disregard of the consumption advisories because the ill effects are long term and relatively unknown to the community unlike the cultural norms (Burger, 2000). Some communities make the risk insignificant by not acting according to the information provided, others follow the guidelines and modify their lifestyle while the rest practice non-adherence to the guidelines (Ashizawa, Hicks, & Rosa, 2005; Bender, 2007; Burger, 2000; Wheatley & Wheatley, 2000).

The Lake Albert fishing community fisheries is a remote fishing community in Uganda whose lifestyle depends on fishing and fishing related activities. This situation, however, is changing due to the discovery and set up of oil wells in the region (Hindrum, 2011). Energy sources as retaliated by Cleveland and others have always had health risks associated with them, the difference being the magnitude and period of onset (Cleveland & Morris, 2014). The health risks are acute and chronic in nature. The acute risks include toxicity and damages or injuries from energy sources like steam, fires, coal, etc. The chronic risks arise from oil spills, fish uptake, and inhalations (Asuquo, Ewa-Oboho, Asuquo, & Udo, 2004; Driscoll, Sorensen, & Deerhake, 2012). Some of the hazards associated with energy sources include mercury and lead (Agusa et al., 2005). These hazards lead to carcinogenicity, neurotoxicity, immune suppression and the cognition effects especially on children under 17 years, women of childbearing age, and pregnant women (Mansilla-Rivera & Rodríguez-Sierra, 2011; Zeilmaker et al., 2013). Both lead and mercury have been established in the fish and fish parts consumed in the Lake Albert community beyond FAO/WHO guidelines (Andrew et al., 2016). Governments have issued fish consumption advisories based on the information available about the hazards quantities in the fish (Engelberth et al., 2013). The gap being addressed is the lack of information about the sociocultural factors affecting fish consumption in the Lake Albert region. This information in turns informs the fish consumption guidelines about the consumption habits and predominant fish consumed. Without a sociocultural study in the Lake Albert, the fish consumption guidelines may not be adopted in a fishing community.

## 2. Methods

### 2.1. Study area

Lake Albert is located in western Uganda, along the following coordinates: 1.52 N; 30.86 E, 10 km deep and occupies the most north part of the rift valley (Karp, Scholz, & McGlue, 2012).
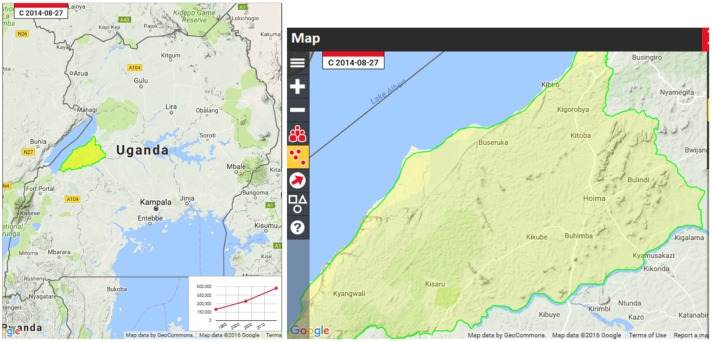


Source: Google (2016).

### 2.2. Household selection

The Lake Albert had four sub counties with landing sites: Kigorobya, Kyangware, Buseruka, and Kabwoya (see Google map). The inclusion criteria involved community households (HH) on and around the Lake Albert landing sites but not more than 2 km from the lake. The members of the selected household must have lived on the landing sites for at least 3–5 years preferably 10 years. Household information was provided by a consenting adult male or female, preferably the household head. The exclusion criteria included households which did not fit the above criteria. A target of 65 households per landing site was randomly selected based on information from local leadership using simple random sampling method by use of random numbers. Sixty-five households per landing site were obtained from a sample size calculation of 260 households which was generated for the whole study (Thrushfield, 1995).

### 2.3. Sample size

The sample size for the households survey is determined by the formula by sample power 3 IBM SPSS software for a population.

*n* = *z*α^2^ *PQ*/*d* ^2^ (Thrushfield, 1995)

*n* = 384 Households

where *n* is the sample size of households, *zα* is the *z* value at *α* = 0.05 level of significance, *P* is the expected prevalence of the condition in the population under study, *Q* is 1 – *P*, and *d* is the desired error of the estimate.

A total of 384 Households is considered sufficient for the study. However, this number was not realized since the households on the four landing sites on Lake Albert were about 500. Therefore, a finite factor for the correction was executed

New sample size = sample size/1 + (sample size − 1/Population) (Thrushfield, 1995)

This gave a new sample size of 217 households. Due to non-responses, an anticipated 20% was factored for the sample size bringing the total respondents to 260. However, the household who participated in the study were 273 and these had the choice of not answering some questions. These were selected randomly from households around the landing sites.

### 2.4. Sampling unit

The sampling unit of the study was the household. The eligibility criteria are that of complete restriction where the household selected is only from those households located around Lake Albert fishing community and inclusion criteria above.

### 2.5. Tools and methods

The household survey involved the use of a structured questionnaire for each of the households. The method of administration of the questionnaire was through semi-structured interviews. This survey was cross-sectional in nature. The interviewers were trained in the administration of the questionnaire. The questionnaires were pretested in a fishing community on Lake Victoria at Kasenyi landing site. After pretesting, the questionnaires were modified for validity and reliability according to the responses generated, and later administered to the respondents (these were either household heads or their spouses) on the Lake Albert landing sites. The major components of the structured questionnaire included: Background information; sociocultural factors (consumption habits, behavior, attitudes, culture and individual factors); activities carried out on the landing sites and information on organizations dealing with monitoring fish and water for heavy metal toxicity (Burger & Gochfeld, 2006; Bushkin-Bedient & Carpenter, 2010).

### 2.6. Data analysis

The data was exported to SPSS version 20 for analysis. The first stage utilized descriptive statistics to generate frequencies and descriptives. The frequencies and descriptives were summarized in tables. An ANOVA analysis was run to compare the amounts of fish consumed weekly with respect to location of the landing site. Then, descriptive statistics using cross tabulations were run to establish associations between factors and the number of times fish is consumed per week. This yielded *χ* ^2^ values whose interpretations were made at the level of significance *α* = 0.05 and confidence intervals of 95%. Those factors which were significant associated to fish consumption were run using the generalized linear model. Generalized linear model analysis using Poisson family was run to depict the contribution of the factors to fish consumption amounts. The generalized linear model output yielded sociocultural factors which aid in the prediction of fish consumption (Valipour, Gholami Sefidkouhi, & Raeini-Sarjaz, 2017).

## 3. Results

### 3.1. Background

**Figure 1.**
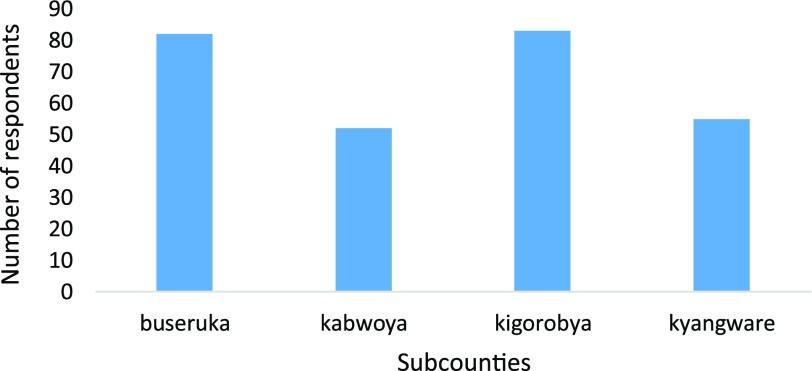
Distribution of study respondents in the four subcounties of Hoima district.

This section of the results shows the demographic parameters related to the households studied in the Lake Albert community. The clustered chart below shows the distribution of respondents per subcounty studied (Figure 1).

The majority (53.3%, *n* = 225) of the respondents lived in the semi urban areas of Hoima district. Most of the respondents (77.7%, *n* = 264) were household heads, ages between 19 and 40 years (64%, *n* = 255), married (71%, *n* = 231), primary education and below (74%, *n* = 262), Christians (87.3%, *n* = 268) and gender, males were 70.7% (*n* = 219). The household member composition ranged from 1 to 20 individuals which is typical of a large African family, with medians of two male and female children and one spouse. The average family size had four members. The rest of the demographic data are shown in Appendix 1.

### 3.2. Consumption data

This section looks at the water and fish consumption data from the households in the Lake Albert community. Over 30% (*n* = 270) of the household obtained their drinking water from the Lake, a place where animals waddle and defecate, boats are washed, and sometimes serves as a toilet for the members on the island. Following up as to whether they boil the water for drinking, 51.1% (*n* = 266) declared that they don’t boil the water for drinking. When asked why they don’t boil the water, 29.9% (*n* = 254) said that the water is safe for drinking without boiling.

Considering fish consumption patterns around the Lake Albert region, 97.4% (*n* = 270) ate fish in all the four landing sites where the study was executed, 91% (*n* = 178) were aware of the benefits associated with fish consumption, the commonly consumed fish species were Tilapia 45.3%, followed by Nile perch 29.8% (*n* = 161), and the major method of preparation was boiling 84.3% (*n* = 236). There was a preference for the parts of the fish consumed depending on whether it was a child less than five year or household head. For children, less than 5 years, 14% (*n* = 268) ate soup while 29% (*n* = 268) were given the middle piece, while for those between 5 and 12 years, the parts given were evenly spread out and the same applied to the teenager (13–18 years) while the adults 33% (*n* = 269) consumed the head. Considering the fish catches from the Lake Albert, pelagic fish were the most highly harvested, followed by Tilapia and Nile perch (34.5, 30.4, and 26.9% *n* = 171), respectively. The average number of times fish is consumed per household in a week is four times and the average amount consumed weekly per household is two kilograms. A unit variance model executed for the Subcounty or location of landing site and the weekly frequency of fish consumed per household revealed an intercept value = 25063, *p* < 0.001; Subcounty value = 57.465, *p* = 0.228. This showed that there was no significant difference between the weekly frequency of fish consumed per household and the landing sites (see Appendix 2).

### 3.3. Activities on landing sites

The most common income generating activities on the landing site are fishing, trade in different commodities and charcoal selling (66.7, 42.5, and 4.76%, *n* = 273), respectively. While the most common recreation activities on the landing sites were bars and hotels followed by sports and games (81.3 and 23.8%, *n* = 273), respectively. The organizations on the landing sites that monitored fishing and other related activities on the landing sites according to the respondents were: Beach Management Unit (BMU), Fisheries department, Enforcement unit and Donor support organization i.e. Republic of Iceland (64.5, 34.2, 13.9, and 0.4%, *n* = 273) (see Appendix 3).

### 3.4. Relationship between sociocultural factors and the amount of fish consumed in the household

This section shows the sociocultural factors related to the weekly frequency of fish consumption in the household, *χ* ^2^, and probability values (*p* values). The probability values are interpreted at (*p* < 0.05). Regarding background, the parameters associated with fish consumption were: household number and the presence of a male child in the home. While for the consumption aspect, it was method of preparation, part of the fish consumed by adults, reasons for eating fish and awareness about the benefits of eating fish that mattered. For activities executed around the landing sites, charcoal selling and recreation facilities were associated with fish consumption amounts. The parameters, *χ* ^2^ and their associated *p* values are displayed in Table 1.

**Table 1.** Relationship between the weekly frequency of fish consumption and sociocultural factors

Attribute	*χ*^2^	Degree of freedom	*p* value
Household number	2,980.6	2,784	0.05
Male child in household	4,258.9	2,808	<0.0001
Awareness of benefits of fish consumption	151	12	<0.0001
Methods of preparation of fish	474.3	60	<0.0001
Parts of the fish consumed by adults	610.2	84	<0.0001
Fish was consumed because it was suitable	139.1	12	<0.0001
Eating of young fish	153.9	12	<0.0001
Charcoal selling	183.3	12	<0.0001
Go to bars/hotels for recreation	181.6	12	<0.0001
Beach management unit	133.7	12	<0.0001
Donor supports like republic of Iceland	153.9	12	<0.0001

### 3.5. Assessing the contribution of the factors to weekly frequency of fish consumption in households

This section shows the contribution made by each factor toward weekly fish consumption frequency in the Lake Albert region. The generalized linear model showed the direction of the response (affirmative or negative) in relationship to fish consumption. The positively correlated factors were households engaged in charcoal selling, household number, presence of a male child in the home, salting as method of preparation of fish, adults’ preference for the fish (Whole fish, Head, Head and Middle piece, and any part of the fish), household who think fish consumption is suitable and awareness about the BMU. While as the negatively correlated factors were households that ate young fish, knowledgeable about a donor organization, awareness about the benefits of eating fish and method of fish preparation i.e. frying. The contribution of the different sociocultural factors is shown in Table 2.

**Table 2.** Contribution of the sociocultural factors to weekly frequency of fish consumption in the household

Factor	Estimate	Std. error	*z* value	Pr(>|*z*|)	Lower confidence interval (2.5%)	Upper confidence interval (97.5%)
(Intercept)	3.48689	0.27411	12.721	<2e-16	2.962399790	4.0305392
charcoalselling [T.1]	1.96128	0.44825	4.375	1.21e-05	1.116670377	2.8614162
eatyoungfish [T.1]	−2.43621	0.79327	−3.071	0.002133	−4.032920511	−0.7780923
iceland [T.1]	−1.98893	0.53609	−3.710	0.000207	−2.896514615	–0.7313155
x.701hhnos	0.07602	0.03834	1.983	0.047384	0.003016785	0.1491753
x.701malechild	0.16529	0.06151	2.687	0.007202	0.051545403	0.2796352
x.901bawareaboutbenefits [T.1]	−1.45466	0.18219	−7.984	1.41e-15	−1.825136911	−1.0883689
x.1002dmethodspreparation [T.frying]	−0.65934	0.21627	−3.049	0.002299	−1.072426866	−0.2315390
x.1002dmethodspreparation [T.salting]	5.59302	0.88848	6.295	3.07e-10	3.829377617	7.5212907
x.1003aadults [T.Whole fish]	2.54979	0.48831	5.222	1.77e-07	1.616426287	3.5496740
x.1003aadults [T.MP]	0.45987	0.27040	1.701	0.088994	−0.069589343	1.0047853
x.1003aadults [T.Any]	0.77940	0.23654	3.295	0.000984	0.311221687	1.2548157
x.1003aadults [T.H,MP]	6.58951	0.97268	6.775	1.25e-11	4.764909534	8.6205241
x.1003breasonssuitable [T.1]	0.78639	0.18399	4.274	1.92e-05	0.418533385	1.1566487
x.1005corganizationsmonitoringbmu [T.1]	1.95863	0.19378	10.107	<2e-16	1.568760925	2.3410450

The GLM output generated is displayed in the model below: glm (formula = x.1002bnumberoftimesweek − charcoal selling + eat young fish + iceland + x.701householdnos + x.701malechild + x.901bawareaboutbenefits + x.1002dmethodspreparation + x.1003aadults + x.1003breasonssuitable + x.1005corganizationsmonitoringbmu, family = poisson (identity), data = Hoima1) Null deviance: 1757.4 on 811 degrees of freedom Residual deviance: 1242.0 on 792 degrees of freedom AIC: 4031.5 Number of Fisher Scoring iterations: 7.

## 4. Discussion

### 4.1. Demographic characteristics if fishing communities

The study area is representative of a rural fishing village in a developing country where most of the household heads are young people, uneducated, married, have large family sizes and low life expectancy (Ssetaala et al., 2012). The majority (64%) of the household heads were aged between 19 and 40 years and married, an attribute of a country with a growing population. Early marriages in Uganda are responsible for the population explosion since each female has a fertility index of about seven children on average, a position the country is trying to modify through the National Population Action Plan (UBOS, 2012). This increasing population strains the already limited resources i.e. fish available for consumption in the community (Majale, 2014). Even when the fish is available, access will be limited due to the high price required for purchase. The rations of fish consumed are reduced leading to loss of benefits conferred through fish consumption (Gimou et al., 2013). This situation shall worsen through consumption of contaminated fish (Tamale et al., 2016a). The contaminated fish will result in high exposures to hazards contained therein leading to fish toxicity cases (Tang, Kwong, Chung, Ho, & Xiao, 2009). This resultant fish toxicity will result into increased mortalities from cancers and nervous disorders, therefore, decreasing the life expectancy of the community especially the pregnant and childbearing age women in Hoima District. These women are enterprising, and their deaths will create a high level of dependence among the orphaned families in Lake Albert (Majale, 2014; Ssebisubi, 2013). Therefore, a fish consumption advisory can be a timely tool to avert this bizarre situation in the rather impoverished Lake Albert (Dahshan, Abd-Elall, & Megahed, 2013).

However, there was limited to no education in the community, an attribute of developing countries (Tamale et al., 2016b). This limited education reduces on the skill sets of the fishing community making them vulnerable to change (Burger & Gochfeld, 2008). The limited education also causes literacy challenges in the community hence the need for an interpreter when disseminating the fish consumption advisory guidelines (Shimshack, Ward, & Beatty, 2007). This limited education will also compromise the uptake and acceptability of the fish consumption advisory message (Engelberth et al., 2013). Therefore, to solve the education dilemma, the messages have to be channeled through pictorial displays.

Although the average household sizes were four, families ranged between 1 and 19 members. Small sized families have larger exposure amounts to fish contaminants if there was an incidence of contamination or pollution (Mansilla-Rivera & Rodríguez-Sierra, 2011; Tang et al., 2009). While as a big family size puts the household under pressure to provide the basic necessities of life i.e. shelter, education, food and clothing (Ssetaala et al., 2012). It is the lack of the basics which make the fishing community socially and economically vulnerable (Ssebisubi, 2013). As is the culture in the fishing community, water for human consumption is obtained from the borehole and lake. What Ssetaala et al. (2012) and others found in Buikwe District was that 50% of the drinking water was from the lake. Sources of drinking water act as vehicles through which contaminants from the lake end up in the diet of man (Begum & Yurdakok, 2010). According to water directorate, in Uganda, over 70% of the residents in Hoima District have access to safe drinking water (Majale, 2014). However, the majority of the inhabitants at the landing sites drank unboiled water from bore holes and lakes. The perception was that this water was safe for drinking. This attitude predisposes the community to water-borne diseases (Ssebisubi, 2013). The most prone segment of the population to water borne diseases are the children (Ssebisubi, 2013; Ssetaala et al., 2012). No wonder, when you look at the under-five mortality figures in the fishing communities in Uganda, diarrhea is the number one killer (UNDP, 2014). Therefore, having other hazards in water i.e. mercury and lead, which affect child development, would be compounding the already bizarre situation (Mansilla-Rivera & Rodríguez-Sierra, 2011). Therefore, community advisory guidelines should incorporate water hygiene practices (Donatuto & Harper, 2008). Otherwise, the achievements of the advisory will be undermined by the deaths from acute water-borne diseases.

In addition to the establishment of the hazards in water, most advisories reports have established the levels of the contaminants/hazards in food sources so as to have a complete picture of the community exposure (Mdegela et al., 2009; Tamale et al., 2016a). The summation of risks (Hazard quotients) from various sources for humans’ lead to computation of the hazard index (Narottam & Zaman, 2013). Therefore, the use of total hazard quotients and hazard index have been adopted to sum up the human risks attributed to hazards uptake through various routes i.e. fish, water and fish parts (Narottam & Zaman, 2013). Fish consumption in fishing communities i.e. Lake Albert community is a lifestyle (Barges, 2008). Fish is reflected as a staple in the diet of the fishing community (Drescher et al., 2014; Ssebisubi, 2013). The study revealed that only 3% consented to not eating fish, and this was in agreement with Burger et al. (2014) found in the Saudi Arabian native community. Fish was consumed almost every other day in varying amounts depending on the household size (Lee et al., 2006). However, fish consumption frequency and amounts have a positive correlation with hazards taken up in the human body (Anderson et al., 2004; FAO/WHO, 2011; Tamale et al., 2016a). Depending on the amount of hazard (s) in the fish, there is a need to either reduce the consumption frequency and amount or to withdraw the highly at risk populations i.e. women of childbearing age, pregnant women, and children less than 17 years from consuming the highly contaminated species. These should eat fish which is less contaminated (Drescher et al., 2014; Mertens, Saint-Charles, & Mergler, 2012; Raissy & Ansari, 2014).

### 4.2. Factors associated with fish consumption

In the Lake Albert community, the factors associated with fish consumption include household size, the presence of a male child in a home and awareness about the benefits related to fish consumption. The other factors include methods of preparation of the fish, parts consumed by adults, income generating activities and knowledge of the monitoring organizations for fishing-related activities. Household numbers dictate the species of fish eaten, the amount, and the hazards taken therein (Lee et al., 2006). The families studied had an average of four children and one pregnant woman and these were considered high-risk groups due to the cognition and fetal abnormalities which result from the uptake of fish contaminated with mercury and lead, (Cassady, 2010). This household sizes of Hoima landing sites were similar to those established in Lake Victoria since both were relatively large, a feature of social and economic vulnerability (Ssetaala et al., 2012).

The presence of a male child in the home and the relation with fish consumption frequency in the home is probably explained by the gender roles executed by various members of the fishing community. In Hoima District, the males are the ones who go out to the lake fishing (Ssebisubi, 2013). The female roles regarding fish include trading and preservation as demonstrated in the fishing villages on Lake Victoria (Ssetaala et al., 2012). Therefore, the more male children a household has, the higher the chances of fish consumption (*p* = 0.007). However, the men tend to ignore the risks associated with fish production and so through the execution of their role of food provision, expose the household to food hazards (Anderson et al., 2004; Ashizawa et al., 2005). Therefore, when setting up fish consumption advisories, the gender roles should be clarified in addition to other individual information (Burger & Gochfeld, 2007).

It was surprising that there was a negative association between those who were aware of the benefits of eating fish and the weekly fish consumption frequency (*p* < 0.0001). This fact emphatically spells out the need for a balanced message with both risks and benefits for eating fish in a fish consumption advisory (Béné et al., 2016; Drescher et al., 2014). Without this balanced message where benefits needed to have come before risks, the Belgian study showed that fish consumption reduced in the short term while eliciting the desired behavior change (consuming less contaminated fish species) in the long term (Verbeke et al., 2008). Another aspect to consider is the mismatch between what the fishing community and fish consumption advisory spell out as benefits of eating fish. The discrepancy in information was observed in the case of New York Bight fisherpersons as discussed by Burger and Gochfeld (2009) who continued the same pattern of fish consumption in spite of the recommendations made by the state authorities.

Most of the respondents (83%) choose a healthier method of fish preparation i.e. boiling over other culinary practices. Using boiling as a standard method of cooking revealed that frying and salting had a significant association with weekly frequency of fish consumption in communities (*p* = 0.002 and *p* < 0.0001 respectively). However, according to Driscoll et al. (2012), Carolina residents used fish preparation as a tool to counteract the amounts of hazards found in fish. Nevertheless, this showed no significant reduction in mercury quantity hence the need for community sensitization. Using oil to prepare fish products is associated with increased risks of heart problems, and yet one of the major benefits of consuming fish is to lessen heart issues in addition to other benefits (Burger & Gochfeld, 2009). This desired benefit probably explains why most of the fishing community probably boil the fish in preference to the frying of the fish. Another study executed in Greece revealed that fried fish had increased concentrations of hazards (Kalogeropoulos et al., 2012). On the other hand, salting was mainly used as a method of preservation rather culinary. The salting was used to extend the shelf life of the fish. This salting practice seems to differ from what is executed on other landing sites of Lake Victoria i.e. 2% to preserve tilapia and Nile perch in Buikwe as documented by Ssetaala et al. (2012). This difference in salt usage is explained by the fact that salt production occurs in Hoima District i.e. Kigorobya Sub-county. Regardless of the method of preservation, fish with hazards will remain a potential route of toxicity for the consumers even when dried as was observed by (Olowu, Adewuyi, & Onipede, 2013) among the Nigerian consumers.

Preference for the parts of the fish consumed by adults on a weekly basis could predispose household members to health risks (El-Sadaawy, El-Said, & Sallam, 2013; Mieiro, Pacheco, Pereira, & Duarte, 2009). The family head not only dictates what fish species to eat at home but also parts consumed by the rest of the household members (Ssebisubi, 2013; Tamale et al., 2016a). In Hoima District, household heads who ate any part were associated with increased weekly frequency of fish consumption (*p* < 0.0001) while those who preferred middle piece were loosely associated (*p* = 0.08). However, it was demonstrated by Williams, Ayejuyo, and Adekoya (2007) that different parts of the fish accumulate varying levels of mercury and lead i.e. the tail accumulates the least. Therefore, the least contaminated fish parts should be recommended for children and pregnant mothers. From the study, most of the children consume soup and the middle piece (15 and 30%, respectively). However, according to Williams et al. (2007), the central portion accumulates high amounts of lead than other parts and can only be consumed by the adults (Tamale et al., 2016a). Therefore, based on this insight, the children under five and pregnant women should be recommended to eat the tail part and not the middle part of the fish. Therefore, fish parts accumulate the hazards in different amounts hence the need to link the quantities in the various parts to human risk (Mieiro et al., 2009; Sary & Mohammadi, 2011; Tamale et al., 2016a).

The reasons fish is consumed in the community as revealed by the respondents is that fish consumption was suitable (*p* = 0.0001). The lack of evidenced-based information about the risks consumed with fish uptake is what makes the fishing community vulnerable to fish hazards (Béné et al., 2016; Loring & Duffy, 2011). Therefore, information about the risks associated with the Nile perch and Tilapia consumption is paramount for these communities. This message is in agreement with (Engelberth et al., 2013) who made it clear when dealing with Maine population that the role of the fish consumption advisory was to bring to the forefront key facts about risks and benefits of the staple diets of the fishing community. Behavior change is the targeted goal for fish consumption advisories i.e. consumption of less contaminated fish (Burger, 2000). The household weekly frequency of fish consumption reduces when young or immature fish is availed (*p* = 0.002). The decrease in fish consumption would be attributed to the fact that juveniles were not eaten due to the small size, lack of taste and preference (Driscoll et al., 2012). Lately, migrants have come to the landing site and incorporated new fishing methods which harvest immature fish especially the Nile perch (Ssebisubi, 2013; Ssetaala et al., 2012). Some fishermen have associated consumption of juvenile fish with ill health in children. These factors aid in the decreased consumption of fish by the fishing community especially when the immature fish are the only ones available for the household and market (Ssebisubi, 2013; Ssetaala et al., 2012). The government of Uganda outlawed consumption of small size juvenile fish. This law is reinforced by the BMU, fisheries department, and enforcement unit (Ssebisubi, 2013). Since the BMU is closer to the people of the landing sites, all the immature fish confiscated from illegal fish are burnt or given freely to the community as food. This act partially explains why the fishing community associate the BMU with household weekly fish consumption (*p* < 0.0001). The fact that the BMU is composed of community fishermen who are vigilant in decreasing use of illegal fishing gear and methods, a resurgence of large and mature fish is now available. This community vigilance is in agreement with (Anderson et al., 2004; Lando & Zhang, 2011) who looked at levels of awareness and knowledge of fishing communities in the USA about hazard toxicity and fish consumption advisories so as to predict take up, utilization and retention of the knowledge. These researchers demonstrated that control of hazard should be at the source using environmentally friendly practices. This control strategy is hard to achieve in the short term hence the need for fish consumption advisories. In conclusion, organizations like BMU will indirectly or directly impact on the uptake of the findings of the fish consumption guidelines in the Lake Albert (Wheatley & Wheatley, 2000).

The level of income almost always determines the food and frequency of consumption of fish in the household (*p* < 0.0001). Surprising the revenue activity associated with fish consumption was not fishing (66.7%) but rather charcoal selling where only 5% of the respondents reported participation. Unfortunately, this activity predisposes the fishing community to more lead as a result of charcoal burning, deforestation challenges and erosion of all the fertile soils into the lake (Hindrum, 2011). Therefore, livelihoods diversification be encouraged for the fishing community i.e. trading and boat transport since these already exist on the landing sites (Olale & Henson, 2013; Ssebisubi, 2013). Therefore, fish consumption guidelines need to incorporate the community livelihood as a factor affecting adoption (Mertens et al., 2012).

Donor organizations around the fishing village reduced the weekly fish consumption in households (*p* = 0.0002). On one of the landing sites visited, the organization set up the infrastructure for the BMU. This support was open to the community to sun dry their products to improve the quality of the fish sold. However, this area is also used by law enforcement and fisheries department as their headquarters. Premise sharing with the legislators made the respondents associate donor organizations with decreased fishing, disuse of certain fishing gear and confiscation of the small sized fish obtained from the lake. Therefore, depending on the community, the advisory should be place-based.

## 5. Limitation and strengths

The key strengths of this work are that being the first of its kind in the region, it will go a long way in informing research about sociocultural factors influencing fish consumption around the fishing villages in developing countries. The research sets up a baseline for a fish consumption advisory for vulnerable community in a developing country. The limitation could be attributed to the use of a structured questionnaire which was standardized for fishing communities in Uganda and not worldwide. However, since the households were randomized, the study can generalize and also have high power for interpretation of the findings.

## 6. Conclusion and recommendations

Based on the study, we can conclude that:

  We found that the predominant fish consumed in the Lake Albert were Tilapia > Nile perch > Pelagic fish.

  The Lake Albert fish consumption guidelines should integrate education, gender, household size, food preparation methods, awareness about regulatory bodies, and income generation activities.

Based on the above, the residents should be sensitized about:

 The risks associated with consumption of unboiled water.

 Why children under five should only be given the tailpiece and not the middle piece.

## Funding

This research was supported by the Consortium for Advanced Research Training in Africa. CARTA is jointly led by the African Population and Health Research Center (APHRC) and the University of the Witwatersrand and funded by the Wellcome Trust (UK) [grant number 087547/Z/08/Z], the Department for International Development (DfID) under the Development Partnerships in Higher Education (DelPHE), the Carnegie Corporation of New York [grant number B 8606], the Ford Foundation [grant number 1100-0399], Google.Org [grant number 191994], Sida [grant number 54100029], MacArthur Foundation[grant number 10-95915-000-INP], and British Council. This research was also supported by Water Network of RIISE Carnegie New York.

## Appendix Appendix 1

### Background information of the Respondents

**Table:**

Category	Attribute	Frequency	Percentage	Sample size
Location	Urban	17	7.6	225
	Rural	88	39.1	
	Semi urban	120	53.3	
Status	HH	205	77.7	264
	Spouse	47	17.8	
	Child	11	4.2	
	Other	1	0.4	
Age	<18	4	1.6	255
	19–30	98	38.4	
	31–40	66	25.9	
	41–50	54	21.2	
	51 and above	33	12.9	
Martial	Single	35	15.2	231
	Married	164	71.0	
	Divorced	10	4.3	
	Widowed	13	5.6	
	Separated	9	3.9	
Education	No formal	38	14.5	262
	Primary	158	60.3	
	Secondary	66	25.2	
Religion	Christian	234	87.3	268
	Muslim	25	9.3	
	Traditionalist	2	0.7	
	Other	6	2.2	

**Water consumption data**

**Table:**

Category	Attribute	Frequency	Percentage	Sample *n*
Source of drinking water	Stream	6	2.2	270
	Bore	104	38.5	
	Spring	1	0.4	
	Tap	59	21.9	
	Lake	82	30.4	
	Other shop	18	6.7	
Amount taken daily	Half liter	5	1.9	270
	One liter	28	10.4	
	1.5 liters	40	14.8	
	2 liters	76	28.1	
	>2 liters	121	44.8	
Where water is kept	Pot	112	43.2	259
	Jellycan	128	49.4	
	Saucepan	2	0.8	
	Plastic container	8	3.1	
	Buys shop	9	3.5	
Whether you boil water	No	136	51.1	266
	Yes	129	48.5	
Major reasons why not boil water for drinking	It is safe	76	29.9	254
	Lacks fire wood	14	5.5	
	No saucepan	4	1.6	
	No time to boil	7	2.8	
	Use water guard	7	2.8	

## Appendix Appendix 2

### Fish consumption amounts

**Table:**

Category	Attribute	Frequency	Percentage	Sample *n*
Eat fish	Yes	263	97.4	270
Awareness about benefits fish	Yes	162	91.0	178
Fish commonly eaten at landing sites	*Lates species*	48	29.8	161
	*Tilapia*	73	45.3	
	Pelagic fish	25	15.5	
Common methods of preparation of fish	Boiling	199	84.3	236
	Frying	32	13.6	
Parts of fish eaten by children less than 5 years	MP	78	29.1	268
	Soup	40	14.9	
Parts consumed by Children between 5 and 12 years	Head	34	12.7	267
	MP	34	12.7	
	T	34	12.7	
	Any	37	13.9	
Fish parts consumed by teenagers	Head	33	12.4	267
	MP	29	10.9	
	Any	42	15.7	
Fish parts consumed by adults	Head	89	33.1	269
	Any	58	21.6	
Reasons for the parts consumed	Suitable for the person	87	31.8	273
	Less bones	114	43.3	263
	Eat young fish	3	1.1	273
Fish commonly captured from lake	Tilapia	52	30.4	171
	Nile perch	46	26.9	
	Pelagic fish	59	34.5	

Notes: T = Tail piece and MP = Middle piece.

## Appendix Appendix 3

### Activities on the landing sites

**Table:**

Category	Attribute	Frequency	Percentage	Sample *n*
Income generation activities	Fishing	182	66.7	273
	Trade	116	42.5	273
	Charcoal selling	13	4.76	273
Recreation activities	Recreation bars/hotels	222	81.3	273
	Sports	65	23.8	273
Monitoring organizations	Beach management unit	176	64.5	273
	Fisheries	96	35.2	273
	Enforcement unit	38	13.9	273
	Donor support i.e. Republic of Iceland	1	0.4	273
